# MicroRNAs Regulating Tumor Immune Response in the Prediction of the Outcome in Patients With Breast Cancer

**DOI:** 10.3389/fmolb.2021.668534

**Published:** 2021-06-09

**Authors:** Konstantina Thomopoulou, Chara Papadaki, Alexia Monastirioti, George Koronakis, Anastasia Mala, Despoina Kalapanida, Dimitrios Mavroudis, Sofia Agelaki

**Affiliations:** ^1^ Department of Medical Oncology, University General Hospital, Crete, Heraklion, Greece; ^2^ Laboratory of Translational Oncology, School of Medicine, University of Crete, Heraklion, Greece

**Keywords:** circulating miRNAs, early breast cancer, metastatic breast cancer, immune response, prognosis

## Abstract

MicroRNAs (miRNAs) are key regulators in immune surveillance and immune escape as well as modulators in the metastatic process of breast cancer cells. We evaluated the differential expression of plasma miR-10b, miR-19a, miR-20a, miR-126 and miR-155, which regulate immune response in breast cancer progression and we investigated their clinical relevance in the outcomes of breast cancer patients. Plasma samples were obtained from early (eBC; *n* = 140) and metastatic (mBC; *n* = 64) breast cancer patients before adjuvant or first-line chemotherapy, respectively. Plasma miRNA expression levels were assessed by qRT-PCR. We revealed a 4-miRNA panel consisted of miR-19a, miR-20a, miR-126, and miR-155 able to discriminate eBC from mBC patients with an AUC of 0.802 (*p* < 0.001). Survival analysis in eBC patients revealed that low miR-10b and miR-155 expression was associated with shorter disease free survival (disease free survival; *p* = 0.012 and *p* = 0.04, respectively) compared to high expression. Furthermore, miR-126 expression was associated with shorter overall survival (overall survival; *p* = 0.045). In multivariate analysis the number of infiltrated axillary lymph nodes and low miR-10b expression independently predicted for shorter DFS (HR: 2.538; *p* = 0.002 and HR: 1.943; *p* = 0.033, respectively) and axillary lymph nodes and low miR-126 for shorter OS (HR: 3.537; *p* = 0.001 and HR: 2.558; *p* = 0.018). In the subgroup of triple negative breast cancer (TNBC) patients, low miR-155 expression independently predicted for shorter DFS (HR: 5.056; *p* = 0.037). Accordingly in mBC, patients with low miR-10b expression had shorter progression free survival and OS compared to patients with high expression (*p* = 0.0017 and *p* = 0.042, respectively). In multivariate analysis, recurrent disease and low miR-10b expression independently predicted for shorter PFS (HR: 2.657; *p* = 0.001 and HR: 1.920; *p* = 0.017, respectively), whereas performance status two independently predicted for shorter OS (HR: 2.031; *p* = 0.03). In summary, deregulated expression of circulating miRNAs involved in tumor and immune cell interactions evaluated before adjuvant and 1^st^-line chemotherapy can distinguish disease status and emerge as independent predictors for outcomes of breast cancer patients.

## Introduction

Metastatic dissemination remains the main cause of morbidity and mortality in patients with breast cancer (Riggio et al., 2021). Despite the progress in diagnosis and treatment of early breast cancer, about 25% of patients are still at high risk for developing metastases in distant organs, whereas the survival rates of those with metastatic disease have only modestly improved during the last years (Caswell-Jin et al., 2018). Therefore, there is an unmet need for novel treatment strategies and for the identification of biomarkers for better stratification of patients according to the risk of recurrence and disease progression.

Immune system recognizes and destroys cancer cells through specific antigens on their surface (Schreiber et al., 2011). However, cancer cells escape from anti-tumor immune response and create a pre-metastatic environment that allows them to proliferate and invade (Kitamura et al., 2015). According to the cancer immunoediting theory the immune system can influence tumor development through a three-step process: elimination, equilibrium and escape (Schreiber et al., 2011). During the first two phases, cancer cells are eliminated by the immune system which results in the prevention of tumour growth. Under the constant selective pressure from the immune system, cancer cells acquire genetic and epigenetic alterations that allow them to grow despite the ongoing immune response (Schreiber et al., 2011). Resistant clones selected through the equilibrium phase avoid detection and eradication by the immune system through multiple mechanisms to enable tumor progression and metastasis (Dunn et al., 2002). Specifically, tumor cells modulate the recruitment of tumor-associated macrophages (TAMs), T regulatory cells (Tregs) and myeloid-derived suppressor cells (MDSc) in the tumour microenvironment (Kitamura et al., 2015), thus suppressing the cytotoxic function of natural killer (NK) cells and CD8+ T cells through the expression of molecules such as programmed cell death ligand 1 (PDL1) and promoting tumor survival and metastatic potential (Kitamura et al., 2015).

Although breast cancer was not traditionally considered as an immunogenic tumor, tumor-infiltrating lymphocytes (TILs) have been consistently documented in breast cancer and have been associated with favourable prognosis in patients with triple negative breast cancer (TNBC) and HER2 positive (HER2+) breast cancer (Loi, 2013). The majority of TILs in cancer are of the T-cell phenotype, which includes CD4+ (helper cells) and CD8+ (cytotoxic cells) lymphocytes, and it has been consistently shown that the presence of CD8+ T-lymphocytes in ER and HER2+ breast cancer are correlated with better clinical outcomes (Ali et al., 2014). TAMs, constitute a prominent component of the tumor microenvironment (TME) in breast cancer. Macrophages exhibit high plasticity in response to TME signals such as interferon and interleukin 4 (IL-4), and polarize either to the pro-inflammatory, M1-like phenotype or to the immunosuppressive, pro-tumor M2-like phenotype to restrict or support primary tumor growth and metastatic spread, respectively (Martinez and Gordon, 2014). In a recent meta-analysis with a total of 4,541 breast cancer patients, high TAMs infiltration was significantly correlated with aggressive clinicopathological characteristics and poor patient’s outcome (Zhao et al., 2017; Jeong et al., 2019). NK cells are the natural guards of the innate immune system and represent important mediators of tumor immunosurveillance and eradication (Vivier et al., 2008). It has been recently shown that NK cells are abundant early responders to disseminated breast cancer cells which must overcome NK cell surveillance in order to form distant metastases (Chan et al., 2020).

MicroRNAs (miRNAs) have emerged as critical regulators in the interplay among cancer and immune cells (Chakraborty et al., 2020). miRNAs are a class of non-coding RNAs of approximately 20–22 nucleotides, which regulate gene transcription in epigenetic manner by binding to the 3’ untranslated region (UTR) of the mRNA target (Ling et al., 2013). Their expression is deregulated in cancer and they can act either as oncogenes or tumour suppressor genes, regulating tumorigenesis, cell proliferation, apoptosis and metastasis (Zhou et al., 2017).

Circulating miRNAs found in the peripheral blood have attracted considerable interest as non-invasive biomarkers in breast cancer diagnosis and prognosis (Wang et al., 2018). Interestingly, miRNAs are found to be key modulators of both innate and adaptive immune response, controlling the development, and the differentiation of several immune cells (Chandan et al., 2019). Furthermore, miRNAs are critical regulators of immune and cancer cell interactions in the TME controlling both the pro- and anti-tumor immune responses (Cortez et al., 2019). It has been also shown that aberrations in miRNA expression promote carcinogenesis and metastasis (O'Connell et al., 2010b).

Several studies have shown that the cytotoxicity of NK cells is impaired by miR-10b and miR-20a which target the ligands of NKG2D receptor, MICA/MICB, a MHC class I molecule that is expressed on the surface of cancer cells (Tsukerman et al., 2012; Yang et al., 2015). In breast cancer, miR-19a was found to be critical in the polarization of TAMs to M1-like phenotype, through targeting multiple oncogenes such as VEGF and STAT3, preventing breast cancer metastasis (Wang et al., 2010b). On the same direction, miR-126 is able to reshape the TME by promoting the immune surveillance and preventing metastasis, by targeting the stromal cell-derived factor-1a, SDF-1A, and by suppressing the chemokine C-C motif ligand 2 (CCL1) (Zhang et al., 2013). Both of these cytokines promote tumor invasion and metastasis through the activation of mesenchymal stem cells and monocytes (Qian et al., 2011). In mouse models, knockdown of miR-155 significantly accelerated tumor growth by impairing the activation of M1-like macrophages (Zonari et al., 2013).

We have previously shown that the above miRNAs are differentially expressed among healthy women and eBC patients (Papadaki et al., 2020b). In addition, a differential expression pattern was encountered among relapsed and non-relapsed patients with early disease (Papadaki et al., 2020b). Based on the above evidence indicating an association of these miRNAs with disease progression, we hypothesized that the expression of miR-10b, miR-19a, miR-20a, miR-126, and miR-155 in the plasma may differ among patients with eBC and mBC and that their expression is associated with significant prognostic implications in the early and/or metastatic disease stage.

## Materials and Methods

### Patients’ Characteristics and Sample Collection

**FIGURE 1:**
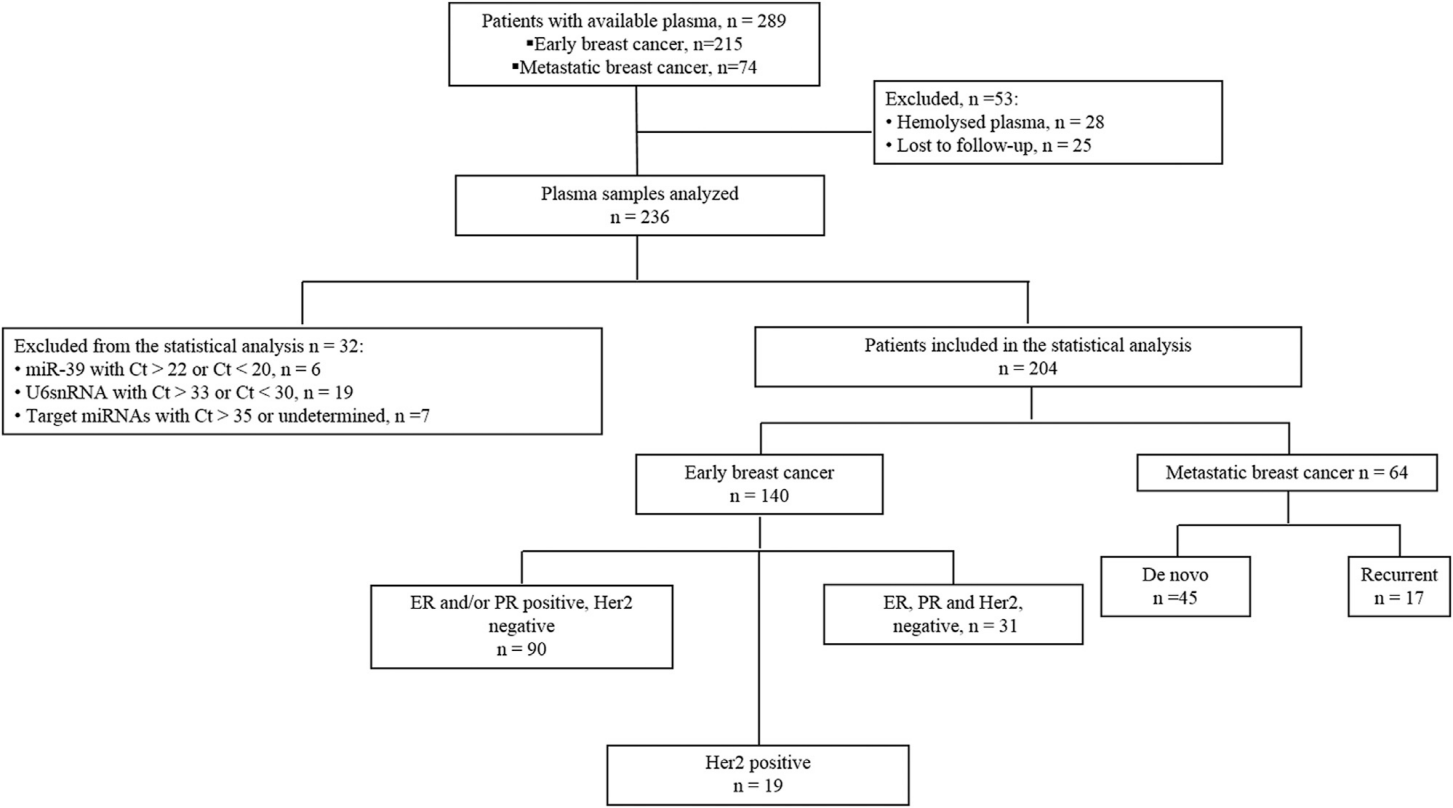
Flow chart of the study. Ct, cycle threshold.

Two-hundred-fifteen patients with eBC who underwent surgery followed by the administration of adjuvant therapy at the Department of Medical Oncology, University Hospital of Heraklion (Crete, Greece) between 2004 and 2011 and available plasma were identified from the clinic records (Figure 1. Blood was collected after surgery and before the administration of adjuvant treatment. Furthermore, 74 patients with metastatic breast cancer (mBC) treated with first-line chemotherapy during the same period in our Institution were also retrieved (Figure 1). Plasma samples were obtained before the initiation of first line chemotherapy.

Also, blood was collected from 20 healthy women to serve as a control group for miRNA relative quantification analysis. Blood was obtained during the procedure of volunteer blood donation performed in the Blood Bank Department of the University General Hospital of Heraklion. The median age of healthy women was 53 years (range 35–60). All patients and healthy donors had signed an informed consent to participate in the study which was approved by the Ethics and Scientific Committee of the University Hospital of Heraklion (ID 2029; Crete, Greece). Clinical characteristics and follow-up information for each patient were prospectively collected. Data cut-off date was December 20, 2019. Peripheral blood from patients and healthy donors was drawn early in the morning and was collected in EDTA- tubes. In patients, blood samples were obtained the same day before starting adjuvant or first line treatment. Plasma was subsequently isolated within 2 h by centrifugation at 2,500 rpm for 15 min at 4°C, followed by a second centrifugation in 2,000 g for 15 min at 4°C to remove cellular debris. Samples were kept in aliquots at −800C until further use. Plasma samples presenting a change of colour to pink (*n* = 28), suggesting the presence of hemolysis, were not processed for further analysis (Figure 1). Furthermore, plasma samples from patients lost to follow up (*n* = 25) were not processed for further analysis as well (Figure 1).

### MicroRNA Expression Analysis

#### RNA Isolation

Trizol LS was used for RNA extraction from 400 μl plasma (Ambion, Life Technologies) as described previously (Papadaki et al., 2018). Briefly, following denaturation by Trizol LS, 25 fmoles of the synthetic *C. elegans* miRNA, cel-miR-39 (Qiagen GmbH, Hilden, Germany) were added in each sample to serve as an exogenous control. Chloroform was added for phase separation and after centrifugation, an equal volume of 700 μl of aqueous phase from each sample was precipitated by adding 0.7 volumes of isopropanol and 1 μl of glycogen (Qiagen). RNA pellet was resuspended in 50 μl RNAse-free water. RNA from all samples was kept at −80°C until further use in the subsequent cDNA synthesis step.

#### Quantitative Real-Time PCR Analysis of MicroRNA Expression

cDNA synthesis and RT-qPCR was performed using TaqMan technology according to manufacturer’s instructions and as previously described (Mitchell et al., 2008; Papadaki et al., 2019). Stem-loop specific primers for each miRNA were used for reverse transcription (assays ID for each miRNA are provided in Supplementary Table S1; Applied Biosystems, Foster City, CA, United States) in a 5 μl reaction. RT-qPCR was performed in a ViiA seven Real-Time PCR System (Applied Biosystems, Foster City, CA, United States). All experiments for each assay were carried out in triplicate wells. Appropriate negative controls were used in both reverse transcription and RT-qPCR reactions where RNA input was replaced by H_2_O and no template control was used, respectively. Ct values and standard deviations for all the examined miRNAs in each group of patients and in healthy donors are shown in Supplementary Table S2.

Fold change (log10) of each miRNA expression relative to the reference gene U6 snRNA was calculated using the 2-ΔCt method. The expression levels of each target miRNA relative to miRNA expressed in healthy controls was calculated using the 2-ΔΔCt method (Livak and Schmittgen, 2001). The suitability of U6 snRNA as a reference gene was supported by the fact that 1) it was stably and reproducibly expressed among patients and healthy donors (Supplementary Figure S1) and 2) ΔCt between target miRNAs and U6 snRNA was low, demonstrating a similar range of expression.

Samples with mean Ct > 35 or not amplified for target miRNAs (*n* = 7) and samples with mean Ct > 22 or Ct < 20 of cel-miR-39 (*n* = 6), suggesting inefficient RNA extraction, were excluded from the statistical analysis (Figure 1). Finally, samples with mean Ct > 33 or Ct < 30 of U6 snRNA (*n* = 19) were also excluded from the statistical analysis (Figure 1).

### Statistical Analysis

Statistical analysis was performed by the statistical package of the social sciences (SPSS) software, version 22.0 (SPSS Inc. Chicago IL). Patients were divided as high and low expression groups according to the median values (above or equal and below to the median values, respectively). Differential expression was evaluated by Mann-Whitney test. Receiver operating curves (ROC) were constructed and area under the curve (AUC), sensitivity and specificity were calculated to evaluate miRNAs discriminatory performance. Binary logistic regression analysis was performed to identify the best discriminating combinations of miRNAs with clinicopathological parameters. Statistical significance was set at *p* < 0.05 (two-sided test). This report is written according to the Reporting recommendations for tumor marker prognostic studies (REMARK criteria) (McShane et al., 2005).

## Results

### Patients’ Characteristics and Study Design

Flow chart of the study and clinicopathological characteristics of early (*n* = 140) and metastatic (*n* = 64) breast cancer patients are presented in Figure 1and Table 1, respectively. In eBC, the median age was 55 years (range, 27–82 years) and after a median follow-up period of 108.3 months (range, 5.57–182.26 months), 94 (67.1%) patients remained disease-free and 46 (32.9%) had experienced relapse (Table 1). Furthermore, 31 (22.1%) had triple negative breast cancer (TNBC; Figure 1 and Table 1). In the mBC group (Table 1), the median age was 60 years (range, 30–82), 45 (70%) patients had *de novo* metastatic disease and 19 (30%) had recurrent disease. The median follow-up period for mBC patients was 34.7 months (range, 2.0–128.0 months).

**TABLE 1:** Clinical and disease characteristics of breast cancer patients.

	Early breast cancer		Metastatic breast cancer	
–	Whole group	Triple negative	–	
Characteristic	n (%)	n (%)	Characteristic	n (%)
Number of patients	140	31 (22.1)	Number of patients	64
Age (years)			Age (years)	
Median (range)	55 (27–82)	56 (38–75)	Median (range)	60 (30–82)
Menopausal status			Menopausal status	
Pre	53 (37.9)	12 (38.7)	Pre	27 (42)
Post	87 (62.1)	19 (61.3)	Post	37 (58)
Tumor size (cm)			Performance status	
T1	62 (44.3)	10 (32.3)	0–1	50 (78)
T2	70 (50)	19 (61.3)	2	14(22)
T3	8 (5.7)	2 (6.5)	–	–
Histological grade			Histological Grade	
I	5 (3.6)	–	I/II	27 (42)
II	56 (40)	–	III	33 (52)
III	67 (47.9)	7 (22.6)	Unknown	4 (6)
Lobular	8 (5.7)	23 (74.3)	–	–
Unknown	4 (2.9)	1 (3.2)	–	–
Infiltrated lymph nodes			Stage at diagnosis	
0	60 (42.9)	21 (80.6)	Recurrent	19 (30)
1–3	50 (35.7)	4 (12.9)	*de novo* metastatic	45 (70)
≥4	30 (21.4)	6 (19.4)	–	–
ER status			ER status	
Positive	88 (62.9)	–	Positive	50 (78)
Negative	52 (37.1)	–	Negative	14 (22)
PR status			PR status	
Positive	88 (62.9)	–	Positive	45 (70)
Negative	52 (37.1)	–	Negative	19 (30)
Her2 status			Her2 status	
Positive	19 (13.6)	–	Positive	16 (25)
Negative	121 (86.4)	–	Negative	48 (75)
Adjuvant chemotherapy			First line chemotherapy	
Anthracyclines-based	10 (7.1)	2 (6.4)	Taxane-based	40 (63)
Taxanes + Antracyclines	95 (67.9)	27 (87.0)	Taxanes + Anthracyclines	2 (3)
Taxanes-based	26 (18.6)	2 (6.5)	Anthracycline-based	15 (23)
Others	3 (2.1)	–	Others	7 (11)
No	6 (4.3)	–	–	
Relapse status			Relapse status	
Non-relapse	94 (67.1)	19 (61.3)	Non-relapse	1 (1.6)
Relapse	46 (32.9)	12 (38.7)	Relapse	63 (98.4)
Survival status			Survival status	
Alive	111 (79.3)	22 (71.0)	Alive	9 (14.1)
Dead	29 (20.7)	9 (29.0)	Dead	55 (85.9)
–			Response to treatment	
–			PR	39 (61)
–			SD	10 (16)
–			PD	15 (23)
–			Visceral metastasis	
–			Yes	55 (86)
–			No	9 (14)
–			Non visceral metastasis	
–			Yes	53 (83)
–			No	11 (17)

ER, estrogen receptor; PR, progesterone receptor; HER2, human epidermal growth factor receptor two; PR, partial response; SD, stable disease, PD, progressive disease.

### MicroRNA Expression and Statistical Correlations in Breast Cancer Patients

In eBC group, the percentage of patients with low expression of miR-10b was higher among patients with pre-menopausal compared to post-menopausal status (64.2 *vs* 35.8%; chi-square test, *p* = 0.007). No other significant correlations were observed between miRNA expression and clinicopathological parameters [(age, tumor size, histological grade, number of axillary infiltrated lymph nodes, estrogen receptor (ER), progesterone receptor (PR) or human epidermal growth factor receptor 2 (HER2) status; chi-square test, *p* > 0.05)]. In mBC, no significant statistical correlations were observed among miRNAs expressions and common clinicopathological parameters. Moreover, no associations were revealed among miRNAs expression and response to chemotherapy.

### Differential Expression of MicroRNA and Their Ability to Distinguish Early From Metastatic Breast Cancer Patients

**FIGURE 2:**
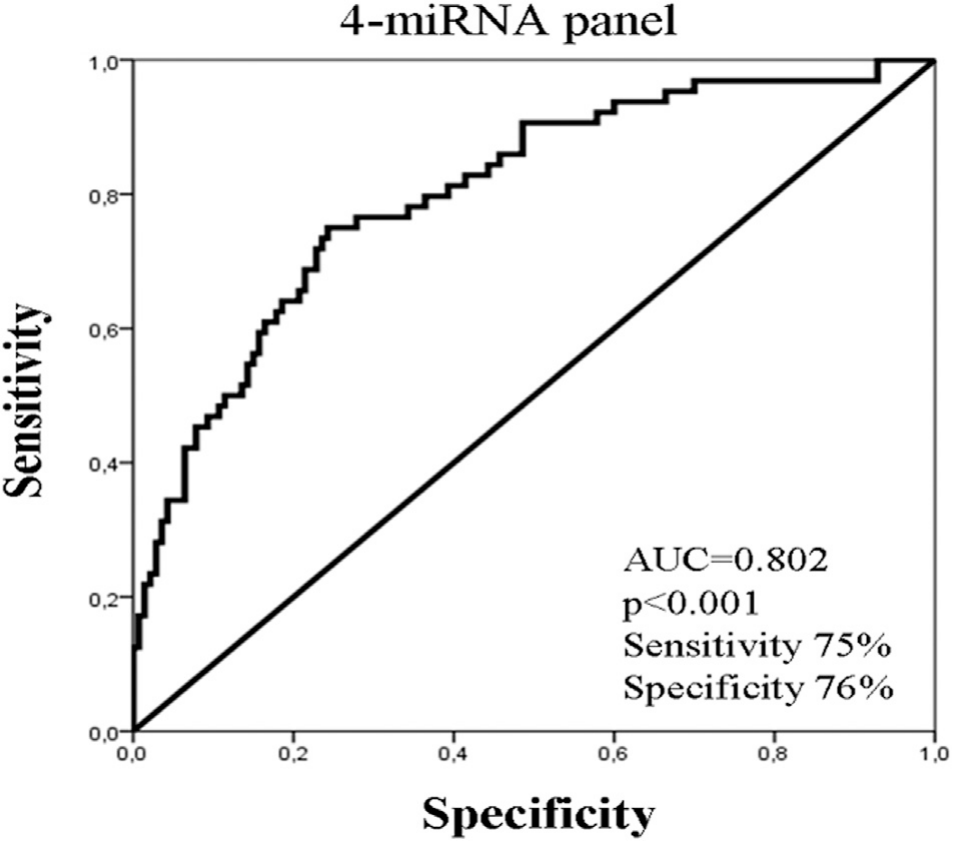
ROC curve analysis depicting the performance of a 4-miRNA panel consisted of miR-19a, miR-20a, miR-126, and miR-155 to distinguish early from metastatic breast cancer patients. AUC, area under curve.

No significant difference in miRNA expression levels were observed among eBC and mBC patients (Mann Whitney test, *p* > 0.05). However, when we assessed the combinations of miRNA expression levels using binary logistic regression, the 4-miRNA panel consisting of miR-19a, mir-20a, miR-126, and miR-155 had the highest performance in distinguishing eBC from mBC patients. The corresponding ROC curve for the 4-miRNA panel showed an AUC of 0.802 (95% CI: 0.728–0.870; *p* < 0.001) with sensitivity of 75% and specificity of 76% (Figure 2)

### MicroRNA Expression and Clinical Outcome in Breast Cancer Patients

**FIGURE 3:**
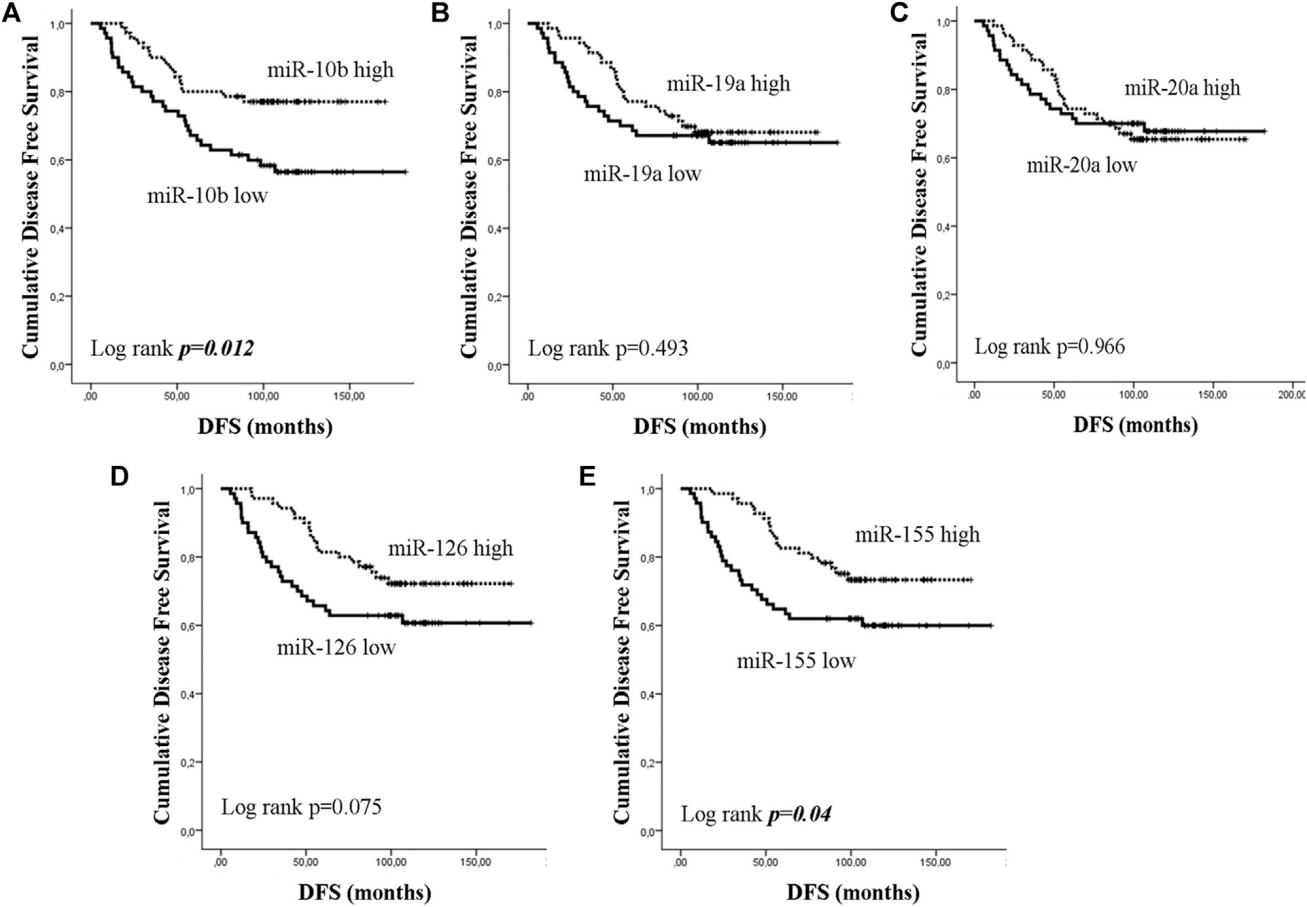
Kaplan–Meier analysis for disease free survival (DFS) according to the expression of circulating miRNAs. Patients were classified as high or low expression groups based on the median value of each miRNA expression. DFS in patients with high or low **(A)** miR-10b, **(B)** miR-19a, **(C)** miR-20a, **(D)** miR-126, and **(E)** miR-155. Curves were compared using the log rank test. P values are shown.

**FIGURE 4:**
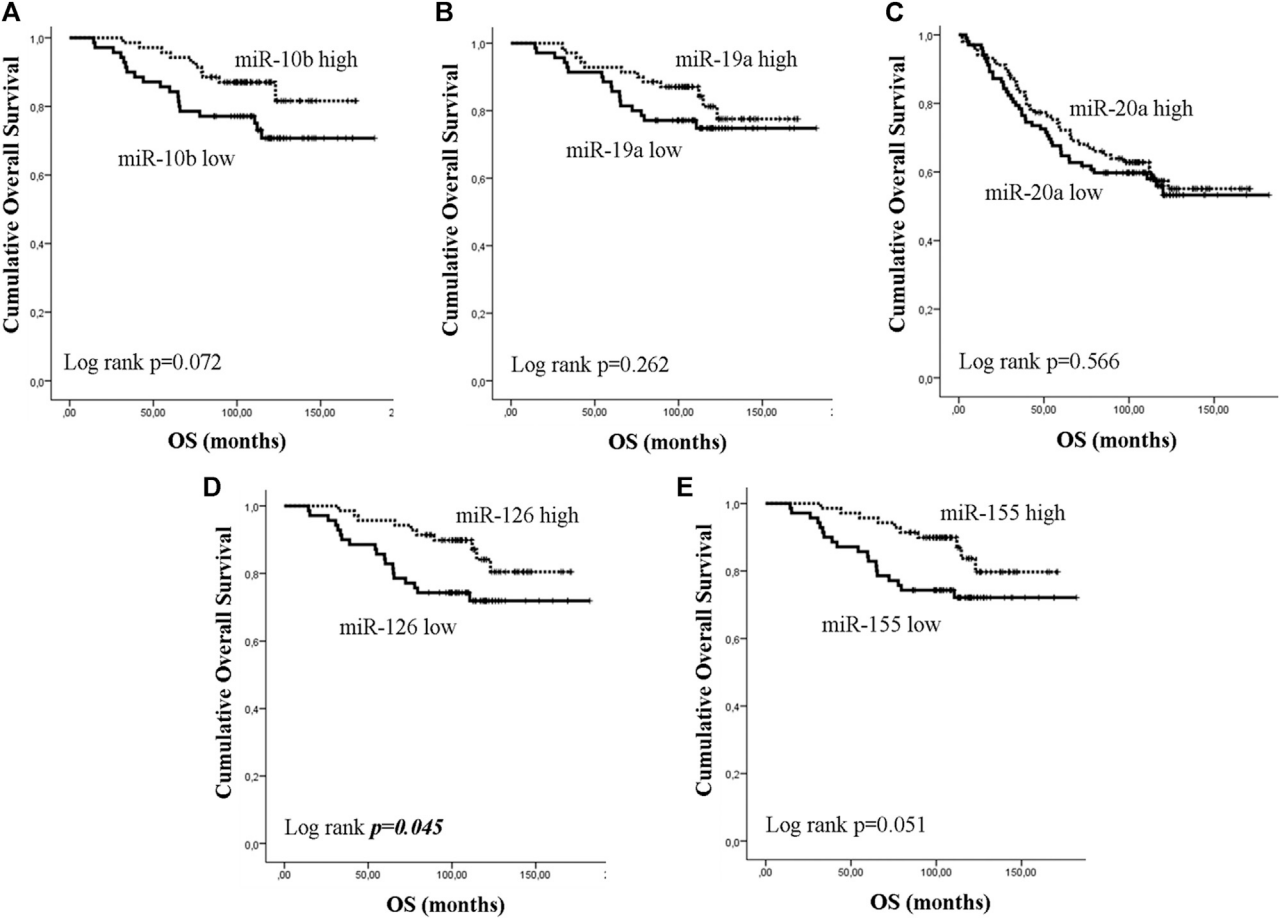
Kaplan–Meier analysis for overall survival (OS) according to the expression of circulating miRNAs. Patients were classified as high or low expression groups based on the median value of each miRNA expression. OS in patients with high or low **(A)** miR-10b, **(B)** miR-19a, **(C)** miR-20a, **(D)** miR-126 and **(E)** miR-155. Curves were compared using the log rank test. *P* values are shown.

Early or metastatic breast cancer patients were divided into two groups with high or low expression according to the median value for each miRNA. In eBC, median DFS or OS were not reached in patients with either high or low expression for any of the investigated miRNAs. However, those with low expression of miR-10b or miR-155 had shorter DFS compared to patients with high expression (both not reached; log rank, *p* = 0.012 and *p* = 0.04, respectively) (Figures 3A,E). No other significant differences were observed among patients with high or low expression for the rest of the miRNAs (log rank, *p* > 0.05) (Figures 3B–D). Furthermore, only patients with low miR-126 had significantly shorter OS compared to patients with high expression (both not reached; log rank, *p* = 0.045) (Figure 4D). Cox univariate analysis, incorporating common clinicopathological characteristics and miRNA expression levels revealed that the presence of more than three infiltrated axillary lymph nodes was associated with decreased DFS and OS (HR: 2.915, 95% CI: 1.618–5.253; *p* < 0.001 and HR: 3.059, 95% CI: 1.470–6.365; *p* = 0.003, respectively) (Table 2) and low miR-10b expression was associated with shorter DFS (HR: 2.134, 95% CI: 1.163–3.916; *p* = 0.014), whereas miR-126 low expression was associated with shorter OS (HR: 2.152, 95% CI: 1.000–4.632; *p* = 0.044) (Table 2). Multivariate analysis revealed the presence of more than three infiltrated axillary lymph nodes as independent predictors for both worse DFS and OS (HR: 2.538, 95% CI: 1.396–4.614; *p* = 0.002 and HR: 3.537, 95% CI: 1,685–7.426; *p* = 0.001, respectively) (Table 2). Also, low miR-10b expression was independently associated with shorter DFS (HR: 1.943, 95% CI: 1.053–3.581; *p* = 0.033) and low miR-126 expression was revealed as an independent predictor for shorter OS (HR: 2.558, 95% CI: 1.177–5.560; *p* = 0.018) (Table 2).

**TABLE 2:** Univariate and multivariate analysis for DFS and OS in patients with early breast cancer (*n* = 140).

Univariate				
–	DFS		OS	
–	HR (95% CI)	*p* Value	HR (95% CI)	*p* Value
Tumor size (T2-T3 vs T1)	1.325 (0.733–2.396)	0.347	1.572 (0.731–3.381)	0.247
Lymph nodes (N3 vs N0-N2)	2.915 (1.618–5.253)	<0.001**	3.059 (1.470–6.365)	0.003*
Histology grade (III vs I/II)	1.808 (0.962–3.400)	0.066	1.237 (0.561–2.726)	0.598
ER (negative vs positive)	1.002 (0.546–1.838)	0.995	1.130 (0.533–2.393)	0.750
PR (negative vs positive)	1.229 (0.680–2.222)	0.496	1.802 (0.869–3.738)	0.113
ER/PR (negative vs at least one positive)	1.206 (0.635–2.292)	0.568	1.629 (0.756–3.508)	0.213
Her2 (positive vs negative)	1.391 (0.648–2.982)	0.568	1.334 (0.509–3.497)	0.558
miR-10b (low vs high)	2.134 (1.163–3.916)	0.014*	1.994 (0.925–4.297)	0.078
miR-19a (low vs high)	1.224 (0.686–2.184)	0.494	1.522 (0.727–3.188)	0.265
miR-20a (low vs high)	1.013 (0.568–1.806)	0.966	1.138 (0.549–2.359)	0.728
miR-126 (low vs high)	1.696 (0.943–3.052)	0.078	2.152 (1.000–4.632)	0.044*
miR-155 (low vs high)	1.708 (0.949–3.074)	0.074	2.111 (0.981–4.542)	0.056
**Multivariate**				
Lymph nodes (N3 vs N0-N2)	2.538 (1.396–4.614)	0.002*	3.537 (1.685–7.426)	0.001*
miR-10b (low vs high)	1.943 (1.055–3.581)	0.033*	–	–
miR-126 (low vs high)	–	–	2.558 (1.177–5.560)	0.018*

DFS, disease-free survival; OS, overall survival; HR, hazard ratio; CI, confidence intervals; ER, estrogen receptor; PR, progesterone receptor; HER2, human epidermal growth factor receptor 2; * *p* < 0.05, **p < 0.001.

**FIGURE 5:**
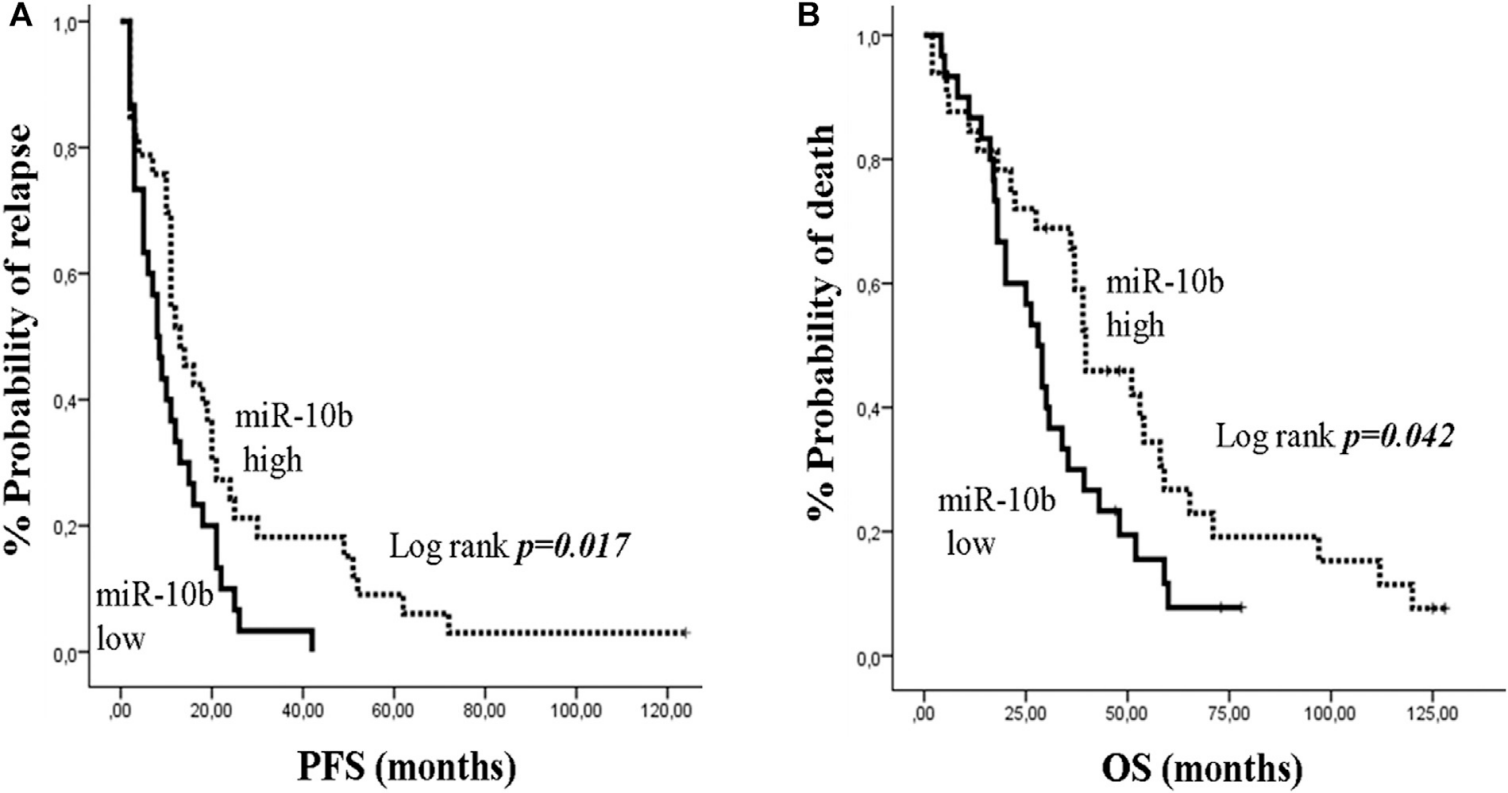
Kaplan–Meier analysis for progression free survival (PFS) and overall survival (OS) according to the expression of circulating miR-10b in metastatic breast cancer patients. Patients were classified as high or low expression groups based on the median value of miR-10b expression. **(A)** PFS and **(B)** OS in patients with high or low miR-10b. Curves were compared using the log rank test. *P* values are shown.

In mBC, median PFS and OS were 11 months (95% CI: 9.06–12.94) and 35.4 months (95% CI: 26.79–44.01), respectively. Kaplan Meier survival analysis showed that only patients with low miR-10b had shorter PFS and OS compared to patients with high expression (13 *vs* 8 months; *p* = 0.017 and 39.7 *vs* 28 months; *p* = 0.042, respectively) (Figures 5A,B). Univariate analysis revealed that recurrent disease and PS 2 were associated with decreased PFS and OS, respectively (HR: 2.544, 95% CI: 1.412–4.584; *p* = 0.002 and HR: 2.031, 95% CI: 1.069–3.860; *p* = 0.030, respectively) (Table 3). In addition, low expression of miR-10b was associated with decreased PFS and OS (HR: 1.845, 95% CI: 1.087–3.131; *p* = 0.023 and HR: 1.756, 95% CI: 1.010–3.082; *p* = 0.046, respectively) (Table 3). In multivariate analysis, recurrent disease and PS 2 independently predicted for shorter PFS and OS, respectively (HR: 2.657, 95% CI: 1.462–4.831; *p* = 0.001 and HR: 2.031, 95% CI: 1.069–3.860; *p* = 0.03, respectively) (Table 3). Also, miR-10b low expression independently predicted for decreased PFS (HR: 1.920, 95% CI: 1.126–3.273; *p* = 0.017) (Table 3).

**TABLE 3:** Univariate and multivariate analysis for PFS and OS in metastatic breast cancer patients (*n* = 64).

Univariate analysis				
Cox regression	PFS		OS	
	HR (95% CI)	*p*-value	HR (95% CI)	*p*-value
Age (<63 vs ≥ 63)	1.46 (0.867–2.458)	0.155	1.169 (0.680–2.010)	0.572
Menopausal status (pre vs post)	1.049 (0.624–1.734)	0.879	1.063 (0.606–1.865)	0.830
PS (2–3 vs 0–1)	1.723 (0.933–3.182)	0.082	2.031 (1.069–3.860)	0.030*
Disease status (recurrent vs *de novo*)	2.544 (1.412–4.584)	0.002*	1.389 (0.778–2.481)	0.266
Grade (III vs I/II)	1.352 (0.800–2.283)	0.260	1.597 (0.898–2.840)	0.111
ER status (negative vs positive)	1.023 (0.546–1.916)	0.944	1.178 (0.619–2.243)	0.618
PR status (negative vs positive)	1.389 (0.798–2.418)	0.245	1.120 (0.623–2.011)	0.705
HER2 (positive vs negative)	1.020 (0.575–1.810)	0.946	1.010 (0.548–1.862)	0.975
Visceral metastasis (yes vs no)	1.485 (0.699–3.153)	0.304	1.143 (0.537–2.436)	0.729
Non visceral metastasis (yes vs no)	1.064 (0.536–2.112)	0.859	1.421 (0.669–3.020)	0.361
miR-10b (low vs high)	1.845 (1.087–3.131)	0.023*	1.756 (1.010–3.082)	0.046*
miR-19a (low vs high)	1.431 (0.865–2.369)	0.163	1.180 (0.688–2.024)	0.548
miR-20a (low vs high)	1.367 (0.826–2.262)	0.224	1.475 (0.860–2.529)	0.158
miR-126 (low vs high)	1.211 (0.735–1.995)	0.453	1.254 (0.731–2.151)	0.411
miR-155 (low vs high)	1.437 (0.868–2.379)	0.158	1.320 (0.760–2.263)	0.314
**Multivariate analysis**				
PS (2–3 vs 0–1)			2.031 (1.069–3.860)	0.03*
Disease status (recurrent vs *de novo*)	2.657 (1.462–4.831)	0.001*		
miR-10b (low vs high)	1.920 (1.126–3.273)	0.017*		

PFS, progression free survival; OS, overall survival; HR, hazard ratio; CI, confidence intervals; PS, performance status; ER, estrogen receptor; PR, progesterone receptor; HER2, human epidermal growth factor receptor; *p < 0.05.

### MicroRNA Expression and Clinical Outcome in Triple Negative Breast Cancer Patients

**FIGURE 6:**
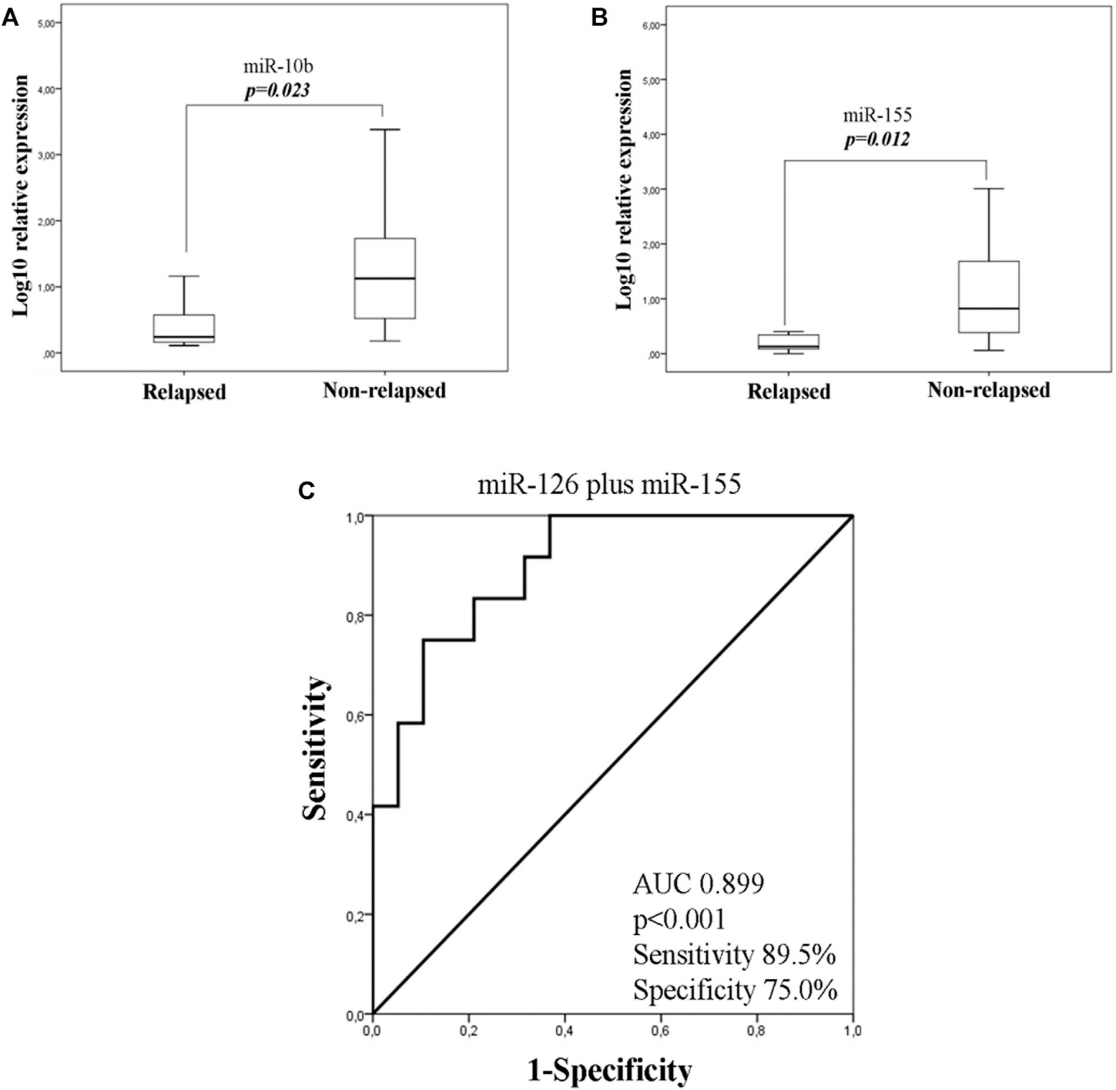
Fold change of miR-10b **(A)** and miR-155 **(B)** in the plasma of TNBC according to relapse status. **(C)**. ROC curve analysis depicting the ability of 2-miRNA panel consisted of miR-126 and miR-155 expression to predict relapsed from non- relapsed patients into TNBC sub-group. TNBC, triple negative breast cancer; AUC, area under curve.

**FIGURE 7:**
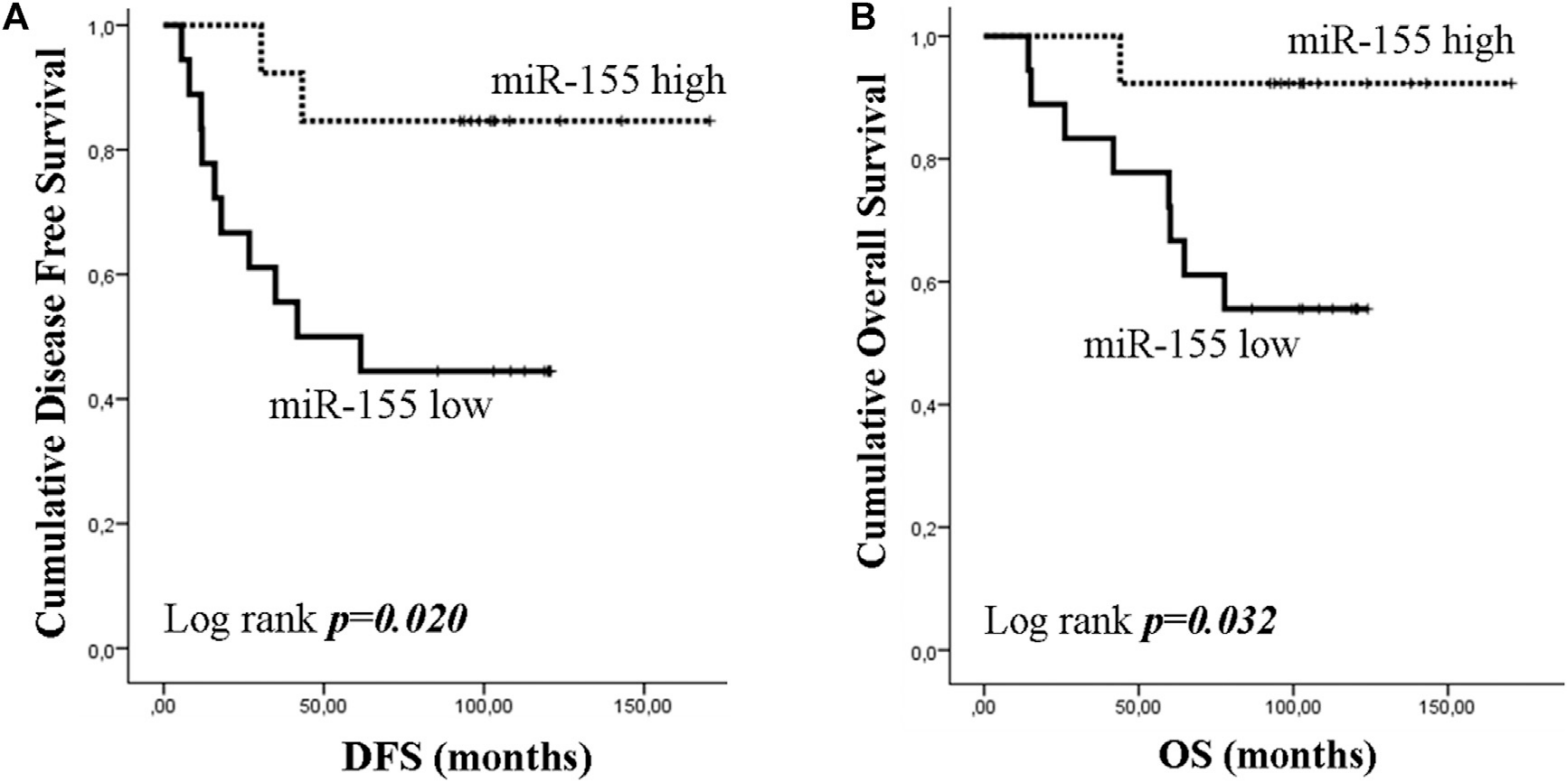
Kaplan–Meier analysis for disease free survival (DFS) and overall survival (OS) according to the expression of circulating miR-155 in triple negative breast cancer (TNBC) group. Patients were classified according to median value of miR-155 expression. DFS **(A)** and OS **(B)** in patients with high or low miR-155. Curves were compared using the log rank test. *P* values are shown.

As TNBC patients have been suggested as more immunogenic, we performed a sub-grouping analysis in these patients. Clinicopathological characteristics are shown in Table 1. The percentage of patients with high expression of miR-19a, miR-126a (both 63.2 *vs* 36.8%; chi-square test, *p* = 0.014) and miR-155 (57.9 *vs* 42.1%; chi-square test, *p* = 0.027) was higher among patients without axillary lymph nodes compared to those with the presence of axillary lymph nodes. No other associations were observed among clinicopathological characteristics and miRNA expression. In addition, no differences were observed in the expression among patients with triple negative breast cancer and patients with other types of receptor status (ER and/or PR+ and HER2+). Differential expression for miR-10b and miR-155 levels was observed among relapsed and non-relapsed patients. Specifically, miR-10b and miR-155 expression levels were lower in relapsed compared to non-relapsed patients (Mann Whitney test, *p* = 0.023 and *p* = 0.012, respectively; Figures 6A,B). When we assessed the combination of the examined miRNAs in binary logistic analysis, a 2-miRNA panel consisted of miR-126 and miR-155 had the highest accuracy to discriminate relapsed from non-relapsed into TNBC sub-group of patients (Figure 6C). In particular, ROC analysis of this combined model revealed an AUC of 0.899 (95% CI: 0.793–1.000) with 89.5% sensitivity and 75.0% specificity (Figure 6C). Finally, patients with low miR-155 expression levels had shorter median DFS and OS (41.73 months *vs* not reached; *p* = 0.02) and not reached; *p* = 0.032, respectively) compared to patients with high expression (Figures 7A,B). In multivariate analysis only miR-155 emerged as independent predictor for shorter DFS (HR: 5.056, 95% CI: 1.104–23.162; p0.037) (Table 4).

**TABLE 4:** Univariate and multivariate analysis for DFS and OS in triple negative subgroup (TNBC) of patients with early breast cancer (*n* = 31).

Univariate				
Cox regression	DFS		OS	
–	HR (95% CI)	*p* Value	HR (95% CI)	*p* Value
Tumor size (T2-T3 vs T1)	1.106 (0.333–3.675)	0.869	1.439 (0.386–5.363)	0.587
Lymph nodes (N3 vs N0-N2)	1.468 (0.397–5.437)	0.565	2.418 (0.603–9.693)	0.213
Histology grade (III vs I/II)	1.175 (0.303–4.552)	0.816	2.519 (0.67–10.120)	0.193
miR-10b (low vs high)	3.040 (0.822–11.246)	0.096	3.391 (0.704–16.341)	0.128
miR-19a (low vs high)	3.232 (0.873–11.965)	0.079	3.274 (0.679–15.774)	0.106
miR-20a (low vs high)	1.013 (0.568–1.806)	0.966	2.415 (0.501–11.633)	0.272
miR-126 (low vs high)	3.132 (0.773–11.005)	0.089	3.274 (0.679–15.774)	0.139
miR-155 (low vs high)	5.056 (1.104–23.162)	0.037*	7.069 (0.883–56.605)	0.065
**Multivariate**				
miR-155 (low vs high)	5.056 (1.104–23.162)	0.037*	–	–

DFS, disease-free survival; OS, overall survival; HR, hazard ratio; CI, confidence intervals; * *p* < 0.05.

## Discussion

The aim of the present study was to explore the differential expression and to evaluate the prognostic significance of immune related miR-10b, miR-19a, miR-20a, miR-126, and miR-155 in the plasma of eBC and mBC patients assessed before adjuvant or first-line chemotherapy, respectively. We found that a 4-miRNA panel consisting of miR-19a, miR-20a, miR-126, and miR-155 could discriminate eBC from mBC patients with high accuracy. We further demonstrate that circulating miRNAs independently predict clinical outcomes in both early and metastatic breast cancer patients.

Mir-10b expression is deregulated in several types of cancer, however the reports regarding its expression profile in breast cancer are controversial (Iorio et al., 2005; Wang et al., 2019). Mir-10b was among the miRNAs found to be down-regulated in primary breast cancer compared to normal tissues (Iorio et al., 2005). In contrast, another report has shown that miR-10b was up-regulated in a small cohort of metastatic breast cancer compared to normal tissue and several preclinical studies suggest a role in invasion and metastasis (Ma et al., 2010; Sheedy and Medarova, 2018). In particular, in a mouse mammary tumor model, silencing of miR-10b significantly increases the levels of its target, Hoxd10 leading to the inhibition of metastasis (Ma et al., 2010). Other studies demonstrate that miR-10b inhibits NK cells to recognize and attack to cancer cells through targeting MICB, a ligand expressed by tumor cells and recognized by NKG2D receptor of NK cells (Tsukerman et al., 2012). Based on the above observation, mir-10b could contribute to immune escape and metastasis through modulation of the immune microenvironment and could be associated to patients’ poor prognosis. Several studies demonstrate that high expression of miR-10b in breast cancer tissues has been correlated with unfavourable pathological parameters and shorter relapse free survival (Parrella et al., 2014; Sheedy and Medarova, 2018). On the other hand limited results exist regarding the role of circulating miR-10b in breast cancer. We herein show that low plasma miR-10b expression was a predictor for shorter DFS in eBC and for shorter PFS in mBC patients. Furthermore results from our lab showed that the expression levels of miR-10b in the plasma were lower in relapsed compared to non-relapsed eBC patients (Papadaki et al., 2020b). Our findings suggest that high miR-10b expression levels in the plasma could reflect effective tumor-immune cell interactions in the TME associated with favourable patients’ outcomes. Taking into account the contradictory results, the role of miR-10b in metastasis needs to be further investigated in eBC.

Mir-155 has a key role in the regulation and function of immune cells (Seddiki et al., 2014). Specifically, mir-155 regulates the differentiation of B-lymphocytes and CD4+ T- lymphocytes and the activation of Tregs (Rodriguez et al., 2007; O'Connell et al., 2010a; Yang et al., 2018). Interestingly, in breast cancer mouse models miR-155 deficiency in DCs impaired their maturation, migration, cytokine production, and their ability to activate T cells (Wang et al., 2016). Accordingly, miR-155 expression is increased upon DC activation during the initiation of the anti-tumor immune response (Wang et al., 2016). Also, down-regulation of mir-155 promoted breast cancer tumor growth by shifting TAMs from the M1-like to the pro-tumour M2-like phenotype (Zonari et al., 2013).

Mir-155 is a well know oncomir, however the results regarding its role in carcinogenesis and tumor progression are controversial (Jiang et al., 2010; Higgs and Slack, 2013). In breast cancer miR-155 functions as an oncomir by targeting the suppressor of cytokine signaling one gene (SOCS1) (Jiang et al., 2010). In contrast, stable expression of miR-155 in 4T1 breast tumor cells reduces the aggressiveness of tumor cell dissemination as a result of preventing epithelial-to-mesenchymal transition (EMT) of tumor cells *in vivo* (Xiang et al., 2011). In the clinical setting, miR-155 expression in TNBC tissues correlated inversely with the expression of several EMT markers while high levels of miR-155 expression was associated with better distant metastasis free survival (Jang et al., 2017). Based on our results, we further show that high plasma miR-155 expression in TNBC patients is associated with better DFS and OS. Interestingly, low miR-155 expression was the only independent predictor for shorter DFS in this subgroup of patients. Furthermore, we showed that circulating low miR-155 expression is associated with shorter DFS in the whole group of eBC patients. Considering the immune regulatory role of miR-155, our findings suggest that higher plasma miR-155 levels could potentially indicate an efficient antitumor immune response. However, other studies show that high expression of serum miR-155 has been correlated with unfavorable clinical characteristics, shorter overall survival and worse disease free survival in eBC patients (Jian Guo et al., 2016). Nevertheless, the prognostic significance of miR-155 needs to be further evaluated in breast cancer.

Preclinical studies have shown that mir-126 act primarily as tumour suppressor by inhibiting cell proliferation and tumor growth (Sun et al., 2010). Also, in TNBC overexpression of mir-126 inhibited proliferation, metastasis and angiogenesis by targeting the regulator of G-protein signaling (RGS3), a gene that is associated with tumour progression and metastasis (Hong et al., 2019). In accordance with preclinical studies, it has been reported that mir-126 expression was low in breast cancer tissues and serum, compared to that of healthy donors (Wang et al., 2010a). Tavazoie et al., suggested that low expression of mir-126 was associated with poor metastasis-free survival in breast cancer patients (Tavazoie et al., 2008). In the same line, we here show that low plasma mir-126 expression is an unfavorable predictor for shorter OS in eBC patients. Furthermore, it has been suggested that high miR-126 prevents metastasis by reshaping tumor microenvironment (Zhang et al., 2013). Specifically, miR-126 represses recruitment of mesenchymal stem cells and inflammatory monocytes, by targeting the stromal cell-derived factor-1a, SDF-1a and the chemokine ligand 2, CCL2, respectively (Zhang et al., 2013). All the above findings come in line with our result and probably the presence of miR-126 in the circulation reflects to a favorable TME.

We further showed that mir-19a and mir-20a included in the predictive model able to distinguish among eBC and mBC patients. These two miRNAs are members of miR-17–92 cluster, which is one of the best-known oncogenic miRNAs, and is overexpressed in multiple cancers such as lung, colon and breast (Olive et al., 2013). Furthermore, recent data have investigated their role as modulators of immune response. Specifically, miR-19a contributes to the shifting of the M2 to M1-like phenotype of TAMs, by inhibiting the proto-oncogene FRA-1 and other downstream genes, such as STAT3 and VEGF (Yang et al., 2014). Several studies suggest a role of miR-20a in immune response, however the results are contradictory. Zhang et al., shows that miR-20a along with miR-17 reduced the suppressive potential of MDSCs by modulating STAT3 expression (Zhang et al., 2011). In contrast, miR-20a targets MICA/B ligands of NKG2D receptor, thus suppressing NK cells cytotoxicity (Xie et al., 2014). Based on our results, mir-19a and miR-20a alone could not be used to discriminate eBC from mBC, however it increased the accuracy of discrimination when used as part of the 4-miRNA panel.

In summary, here we show that the assessment of circulating miRNAs involved in tumor and immune cell interactions, evaluated before adjuvant and first-line chemotherapy, can distinguish disease status and independently predict patient outcomes in breast cancer. Our study is among the first to demonstrate the potential of the investigated miRNAs as non-invasive circulating biomarkers in patients with early and metastatic disease. It has been recognized that miRNAs have multifaceted roles in cancer since the same miRNA may have multiple target genes (Peter, 2010), thus participating in different biological processes. Furthermore, although, differential expression profiles have been demonstrated for circulating miRNAs throughout tumorigenesis and cancer progression (Papadaki et al., 2020a) their biological function remains unclear as yet (Cui et al., 2019). Therefore, we cannot argue that the observed associations between the expression of these miRNAs and survival outcomes are related to the suggested role of these miRNAs in tumor-immune modulation. Deeper investigation is required to unravel the biological function of circulating miRNAs in oncogenesis and tumor progression. Finally, the clinical value of miRNAs as meaningful circulating biomarkers and/or as therapeutic targets merits further investigation in larger cohorts of patients.

## Data Availability

The raw data supporting the conclusion of this article will be made available by the authors, without undue reservation.
