# Corrigendum: MicroRNAs regulating tumor immune response in the prediction of the outcome in patients with breast cancer

**DOI:** 10.3389/fmolb.2024.1413164

**Published:** 2024-04-23

**Authors:** Konstantina Thomopoulou, Chara Papadaki, Alexia Monastirioti, George Koronakis, Anastasia Mala, Despoina Kalapanida, Dimitrios Mavroudis, Sofia Agelaki

**Affiliations:** ^1^ Department of Medical Oncology, University General Hospital, Crete, Heraklion, Greece; ^2^ Laboratory of Translational Oncology, School of Medicine, University of Crete, Heraklion, Greece

**Keywords:** circulating miRNAs, early breast cancer, metastatic breast cancer, immune response, prognosis

In the published article, there was an error regarding the **Affiliations** for Konstantina Thomopoulou. As well as having affiliation 1, they should also have “2 Laboratory of Translational Oncology, School of Medicine, University of Crete, Heraklion, Greece.”

The authors apologize for this error and state that this does not change the scientific conclusions of the article in any way. The original article has been updated.

