# Supplementary material for: MicroRNAs Regulating Tumor Immune Response in the Prediction of the Outcome in Patients With Breast Cancer
Source: Front Mol Biosci. 2021 Jun 9;8:668534. doi: 10.3389/fmolb.2021.668534 (PMC8220200; doi:10.3389/fmolb.2021.668534)
Supplement: Supplementary file 1 [file Table1.DOCX]

Table S1. Assay ID for each miRNA used in the study

| **Name** | **Assay ID** |
| --- | --- |
| hsa-miR-10b-5p | 002218 |
| hsa-miR-19a-3p | 000395 |
| hsa-miR-20a-5p | 000580 |
| hsa-miR-126-3p | 002228 |
| hsa-miR-155-5p | 002623 |
| U6 snRNA | 001973 |
| cel-miR-39-3p | 000200 |
