# Supplementary material for: MicroRNAs Regulating Tumor Immune Response in the Prediction of the Outcome in Patients With Breast Cancer
Source: Front Mol Biosci. 2021 Jun 9;8:668534. doi: 10.3389/fmolb.2021.668534 (PMC8220200; doi:10.3389/fmolb.2021.668534)
Supplement: Supplementary file 2 [file Presentation1.PPTX]

## Slide 1
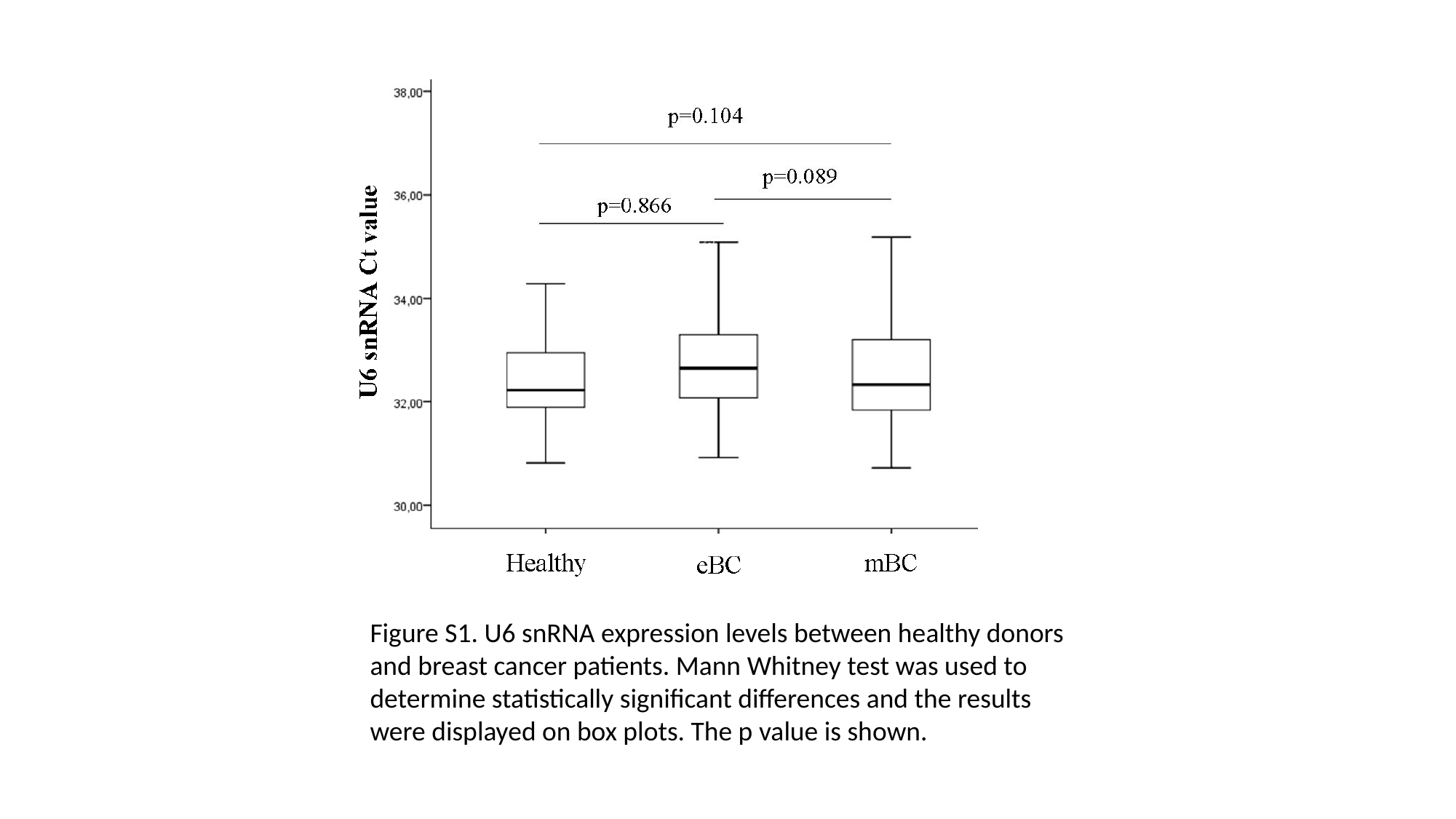

Figure S1. U6 snRNA expression levels between healthy donors and breast cancer patients. Mann Whitney test was used to determine statistically significant differences and the results were displayed on box plots. The p value is shown.
