# Supplementary material for: MicroRNAs Regulating Tumor Immune Response in the Prediction of the Outcome in Patients With Breast Cancer
Source: Front Mol Biosci. 2021 Jun 9;8:668534. doi: 10.3389/fmolb.2021.668534 (PMC8220200; doi:10.3389/fmolb.2021.668534)
Supplement: Supplementary file 3 [file Table2.docx]

Table S2. Median Ct values and SD of each miRNA in healthy donors and breast cancer patients

|  | HD (N=20) | | eBC (N=140) | | mBC (N=64) | |
| --- | --- | --- | --- | --- | --- | --- |
|  | median Ct | SD | median Ct | SD | median Ct | SD |
| miR-10b | 27,00 | ±0,92 | 32.65 | 1.11 | 32.34 | 1.35 |
| miR-19a | 25,56 | ±1,46 | 25.61 | 2.34 | 25.48 | 2.88 |
| miR-20a | 23,93 | ±1,54 | 24.27 | 2.67 | 24.11 | 2.66 |
| miR-126 | 22,83 | ±1,55 | 23.63 | 2.42 | 23.89 | 2.46 |
| miR-155 | 29,07 | ±1,49 | 30.6 | 1.97 | 30.17 | 2.43 |

HD, Healthy donors; eBC, early breast cancer; mBC, metastatic breast cancer
